# Regulation of intestinal growth in response to variations in energy supply and demand

**DOI:** 10.1111/obr.12780

**Published:** 2018-12-03

**Authors:** K. N. Nilaweera, J. R. Speakman

**Affiliations:** ^1^ Department of Food Biosciences Teagasc Food Research Centre Fermoy County Cork Ireland; ^2^ State Key Laboratory of Molecular Developmental Biology, Institute of Genetics and Developmental Biology Chinese Academy of Sciences Beijing China; ^3^ Institute of Biological and Environmental Sciences University of Aberdeen Aberdeen UK

**Keywords:** adipose tissue, hypothalamus, intestine

## Abstract

The growth of the intestine requires energy, which is known to be met by catabolism of ingested nutrients. Paradoxically, during whole body energy deficit including calorie restriction, the intestine grows in size. To understand how and why this happens, we reviewed data from several animal models of energetic challenge. These were bariatric surgery, cold exposure, lactation, dietary whey protein intake and calorie restriction. Notably, these challenges all reduced the adipose tissue mass, altered hypothalamic neuropeptide expression and increased intestinal size. Based on these data, we propose that the loss of energy in the adipose tissue promotes the growth of the intestine via a signalling mechanism involving the hypothalamus. We discuss possible candidates in this pathway including data showing a correlative change in intestinal (ileal) expression of the cyclin D1 gene with adipose tissue mass, adipose derived‐hormone leptin and hypothalamic expression of leptin receptor and the pro‐opiomelanocortin gene. The ability of the intestine to grow in size during depletion of energy stores provides a mechanism to maximize assimilation of ingested energy and in turn sustain critical functions of tissues important for survival.

## Introduction

The intestine is a heterogeneous tissue, containing a number of different cell types including the enterocytes, globlet cells, paneth cells, enteroendocrine cells, M cells and cup cells. These are produced from pluripotent stem cells located at the base of the crypts, by a process of continuous cell division and migration along the crypt‐villi, leading to the attainment of the above differentiated cell fates [reviewed in 1]. Because the intestinal cells have a short‐life span (3–5 d) 2, 3, the cell turn‐over in the gastrointestinal tract is high, accounting for about 20–35% of whole body protein synthesis and likely a substantial contribution to energy expenditure, much of which is linked to the small intestine rather than the stomach or the colon 4, 5. This is not surprising given that the small intestine plays diverse roles including nutrient digestion and absorption, the production of satiety hormones in response to nutrient availability [reviewed in 6, 7, 8]. Given the high energy requirements of the intestine, one can then predict that the energy demands of intestinal tissue growth and maintenance must be via some mechanism linked with energy and nutrient supply to the tissue.

**Figure 1:**
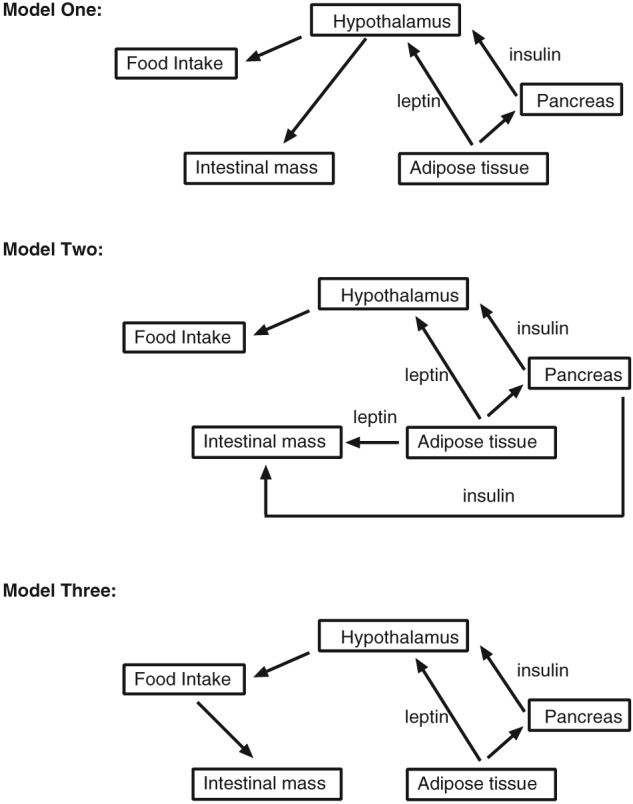
Three different models explaining the potential relationships between adipose tissue, hypothalamus, food intake and intestinal growth. In model one, the reductions in adipose tissue lead to reduced leptin and insulin that pass to the hypothalamus. This brain region then increases food intake and intestinal growth. In model two, the same changes in the adipose tissue lead to elevated food intake via the hypothalamus but directly impact on intestinal growth with no hypothalamic involvement. In model three, the impact on the hypothalamus affects food intake and this directly stimulates intestinal growth.

In this article, we will detail two mechanisms of regulation of intestinal growth. The first is the well‐known mechanism that couples intestinal growth to exogenous nutrient availability. In this process, the intestinal cells directly metabolize dietary nutrients from the gut lumen and use this energy for growth. Another less well‐studied mechanism appears to regulate intestinal growth in response to internal energy reserves, being particularly active during whole body energy deficit. This mechanism paradoxically also increases the intestinal growth, indicating an investment of energy into the intestine during whole body energy deficit. While this physiological change has been shown to occur in mammals, reptiles and birds, we propose here a model of how this happens based on data from five different energetic challenges in mammals. These are bariatric surgery, cold exposure, lactation, dietary whey protein intake and calorie restriction (CR). We propose that a common response to these challenges is that the adipose tissue mobilizes stored fat to sustain critical functions in diverse tissues. We envisage several potential mechanisms by which this depletion of adipose tissue may then be linked to the growth of the intestine (Fig. 1). Adipose tissue produces a large range of secreted hormones (adipokines) in relation to the amount of adipose tissue in the body. The most well known of these is leptin 9, but since its discovery in 1994, there have been many more [reviewed in 10, 11]. Moreover, the amount of stored fat also influences the production of hormones in other tissues such as insulin from the pancreas. In the first model (Fig. 1), the depletion of the adipose tissue mass following energy deficit, signals to the brain, in particular the hypothalamus, a critical region in the brain regulating energy balance, to increase food ingestion and stimulate growth of the intestine to maximize energy assimilation efficiency. Both of these responses would serve to counteract the energy deficit. We propose that the growth of the intestinal tissue occurs alongside the neurobiological drive to consume food, creating an intestinal environment that increases energy absorption from available exogenous nutrient supply. This mechanism would allow the animal to return to energy balance at the earliest opportunity. A second way that this might happen (model two in Fig. 1) is that the secretions from the adipose tissue and other related organs signal to the hypothalamus and this promotes hunger and elevate food intake, but these hormones also have direct impact on intestinal growth, which is not centrally coordinated. Finally, it is possible that there is no ‘second pathway’ and that under energy deficit, the reduced adipose tissue stimulates hunger via the hypothalamus, but it is only in response to the elevated food intake that the alimentary tract grows via uptake of luminal nutrients (model three in Fig. 1).

Given that animals including humans are rarely in energy homeostasis, but rather alternate between periods of energy excess and deficit, depending upon the environmental circumstances (e.g. changes in food availability), highlight a need for regulation of intestinal growth according to energy availability.

## Regulation of intestinal growth from direct utilization of exogenous energy supply in the gut lumen

It is well known that the quantity and quality of dietary nutrients affects the growth and maintenance of the intestine. In this scenario, the intestinal cells use dietary nutrients directly from the gut lumen as an energy source to divide and differentiate into diverse cell fates to replace dying cells [reviewed in 12]. This direct use of nutrients from the gut lumen was demonstrated in the juvenile python (Python molurus), where the surgically isolated central part of the intestine devoid of contact with luminal nutrient did not grow when compared with re‐anastomosed proximal and distal parts that were in direct contact with nutrients 13. Interestingly, the central part showed increased nutrient absorptive capacity without changing tissue mass post‐feeding. In contrast, the re‐anastomosed proximal and distal parts both increased nutrient uptake capacity and tissue mass. Given that the three intestinal regions in the latter study were still in contact with the blood supply and hence circulating nutrients, with only the proximal and distal regions exposed to luminal nutrients, these data suggest that the intestinal cells are able to sense and regulate nutrient uptake both from apical and basolateral sides, whereas the nutrient signals received from the apical side (facing the lumen) appear to promote the growth of the tissue. In line with this suggestion, enteral nutrition as opposed to parenteral nutrition, increased porcine intestine mass 14. Early work from Alpers (1972) further characterized the impact of apical versus basolateral nutrient inputs, by showing that radiolabelled leucine delivered through the lumen was incorporated more into villi‐associated cells, while those delivered intravenously incorporated more into crypt associated cells, possibly by taking advantage of the nutrient supply in the circulatory system 15. The difference in nutrient accessibility along the crypt to villi axis is also reflected in the expression level of nutrient transporters 16, which is driven in part by nutrient load and quality in the lumen 17, 18. This degree of regulation provides a way to absorb and utilize the nutrient‐derived energy as a source of fuel to drive cell division. Notably, the epithelial cells use both the energy sensor AMP‐kinase (AMPK) dependent and independent pathways in this coupling mechanism 19, 20. This latter mechanism also involves mammalian target of rapamycin (mTOR) and the cell cycle‐associated cyclin D1 21. In contrast, the crypt cells use the AMPK and the mTOR to manage energy availability and cell proliferation 22, 23. While more studies are needed to further delineate additional components in these pathways, it is clear that dietary nutrients in the lumen directly act as energy sources for tissue growth and that this provides an explanation as to why over‐nutrition leads to higher cell proliferation, with increased intestinal capacity to absorb energy 24.

## Regulation of intestinal growth during endogenous energy deficit

Because metabolic rate increases with body size 25, the energy required to sustain this from energy absorption must also increase in relation to size. This is achieved in part by larger animals having larger intestines 5, 26. Similarly, environmental factors that impose an energy cost on an animal must ultimately be supplied by elevated energy intake and absorption and hence also often involve growth of the intestine. For example, wading birds in the high arctic have larger intestines than those living at lower altitude 27. The importance of this adaptation for survival is highlighted in the red knot (Calidris canutus), as in the absence of this adaptation, possibly due in part to food shortage in eastern USA during the period between 1997 and 2002, prevented the birds from achieving their ideal body mass needed for migration and breeding in Arctic polar deserts, leading to a reduction in the annual adult survival rates 28. These data suggest that there is a mechanism that couples whole body energy availability with intestinal growth and that imposing an energetic challenge (e.g. living in high latitudes or experiencing food shortage), which affects internal energy availability, causes growth of the intestine to assimilate more energy and counteract the energy deficit (Fig. 1). To understand how information relating to whole body energy availability is conveyed to the intestine, we will explore several challenges that have been shown to cause energy deficits and examine related data to piece together a model of the communication pathway.

### Cold exposure

Because mammals generally regulate their body temperature at euthermic levels, exposure to a cold environment results in elevated energy demands and responses that elevate expenditure and food intake 29. Notably, cold exposure stimulates the growth of brown adipose tissue 30 and conversion of beige adipocytes from their white to brown phase in rats, mice and hamsters following inputs received from hypothalamic neurons [reviewed in 31 and 32]. This allows more fatty acids to be catabolized in the brown and beige adipocytes to generate heat 33. The energy expended to sustain body temperature is replenished by a parallel increase in energy intake, arising from the activity of hypothalamic AMPK 34, along with increased hypothalamic expression of the orexigenic neuropeptides, namely, melanin concentrating hormone and neuropeptide Y (NPY), albeit the effect on latter gene expression has not consistently been shown 35. In relation to anorexigenic neuropeptides, the expression of leptin receptor (Ob‐r) is up‐regulated in the hypothalamus 36, and gene knockout studies show roles for pro‐opiomelanocortin (POMC) and cocaine‐amphetamine regulated transcript (CART) in mediating some of the effects of cold exposure 36, 37, 38. A functioning leptin signalling system is necessary for the increase in food intake in response to cold exposure but not the increased thermogenesis 39, 40. Beyond the hypothalamic changes driving elevated energy intake, cold exposure also increases intestinal weight and energy assimilation 41, 42 (Table 1), which together suggests a potential link between energy loss (either from the lean or white adipose tissue compartments), hypothalamic neuropeptide changes and the intestinal growth acting to assimilate more energy. These changes are consistent with all three of the models presented in Fig. 1.

**Table 1:** Effect of energetic challenges on energy assimilation efficiency or intestinal nutrient absorptive capacity

Energetic challenge	Species	Effect on the energy assimilation (EA) and energy assimilation efficiency (EAE) or nutrient absorption capacity
Cold exposure	Mice	Approximately 2.3‐fold higher EA in males (146.5 ± 10 kJ d^−1^ at −5°C vs. 62.4 ± 7.3 kJ d^−1^ at 22°C) and 2.5 higher EA in females (140.5 ± 7.7 kJ d^−1^ at −5°C vs. 55.9 ± 6.3 kJ d^−1^ at 22°C). This is based on energy consumed and lost in faeces and taking into account 3% energy loss in urine 41.
	Ducks	*In vitro* analysis of ileal sections from cold exposed animals revealed eightfold increased glucose uptake (32 ± 14 vs. 4 ± 1 nmol of D‐glucose per minute and per centimetre tissue at 4°C vs. 25°C, respectively) 42.
Surgery	Humans	Approximately 1.8‐fold lower EA 14 months after RYGB (1,917 ± 156 kcal d^−1^) compared with values before RYGB (3505 ± 217 kcal d^−1^) 54.
	Python	Increased absorption of L‐leucine and L‐lycine by the middle third of the intestine (devoid of contact with luminal nutrients) in resected compared with intact intestine as determined by *in vitro* analysis of the tissues from 6‐d post‐fed animals 13.
	Rats	*In vitro* assays of the distal part of 80% resected intestine of rats exposed to cold (5°C) show increased glucose uptake capacity per milligram of tissue compared with unresected controls exposed to the same temperature 55.
Lactation	Mice	At room temperature, EAE was 81 ± 0.57% in non‐reproductive and 80.8 ± 0.35% in lactating MF1 female mice (*P* = 0.77) 65. The EAE was unaffected by time of reproduction (last week of pregnancy vs. days 6 or 13 of lactation) and was similar to time matched non‐reproductive controls 73.
		In cold exposed MF1 mice, the mean EAE was 79.8 ± 1.17% for non‐lactating females and 82.2 ± 1.01% for the lactating females (*P* = 0.39) 76.
		EAE of shaved and unshaved lactating and control MF1 mice did not differ significantly (lactating shaved [79.9 ± 1.8%] and unshaved [79.8 ± 1.2%] *P* = 0.84; non‐reproductive shaved [78.9 ± 1.4%] and unshaved [78.8 ± 1.5%] *P* = 0.90) 120.
		Lactating MF1 mice with access to a cold area had greater EAE (87.2 ± 1.9%) than those with no access (82.4 ± 0.9%) (*P* = 0.006) 121.
		Increased (39–63%) glucose uptake capacity of the intestine with reproductive demand (pup number) as revealed by *in vitro* analysis of the tissue 122.
	Hamsters	Lactation increased (by approximately 10%) digestive efficiency and intestinal activity for maltase (by 106%), sucrose (by 114%) and aminopeptidase (by 116%) compared to non‐lactating controls 56.
	Brandt's voles	Duration of lactation with variation in temperature (30°C or 21°C) did not affect EAE 74.
	Bank voles	EAE lower in lactating shaved voles than in unshaved ones (shaved: 78.8 ± 0.5%, unshaved: 80.3 ± 0.4%, *P* = 0.036) 123.
Dietary whey proteins	Mice	Excreted fat increased 84.
	Humans	*In vivo* data show a reduced absorption of docosahexaenoic acid, docosapentaenoic acid, eicosapentaenoic acid, alpha‐hydroxydecanoinc acid, lauric acid and myristic acid 80.
		*In vivo* data show increased absorption of tryptophan (sixfold), leucine, valine, lysine and threonine 80.
Calorie restriction (CR)	Monkey	Lower EAE following CR (CR; 91.0 ± 3% vs. 95.0 ± 2% in the controls; *P* < 0.001) 91.
	Rats	The EAE did not differ 90. Digestible efficiency of crude proteins increased (2%) with no change in efficiency for fat or carbohydrates 88, 89.
	Mice	EAE was 2% higher on average in mice under CR between 10% and 40% CR 85.
		*In vitro* data show that CR increases intestinal capacity to absorb D‐glucose (by 28–50%), D‐fructose (by 50–55%), L‐proline (by 44–55%) and L‐glutamine (by 160%) 92, 93.

Related references are in parentheses.

### Bariatric surgery

The Roux‐en‐Y gastric bypass (RYGB) surgery is a commonly used weight loss intervention 43. In this procedure, the stomach size is reduced to a small pouch and is linked to the distal intestine through the ROUX and common limbs. The surgery causes rapid delivery of nutrients to the distal intestine, which increases the production of satiety hormones such as glucagon‐like peptide (GLP)‐1 and peptide YY (PYY) as well as the production of bile acids [reviewed in 44, 45]. This surgical procedure decreases energy intake, which, together with increased sympathetic nerve stimulation of peripheral tissues 46, reduces the body and fat weight 47. Interestingly, the targeted deletion of the anorexigenic melanocortin‐4 receptor (MC‐4R) gene in mice partially prevented the loss of body weight associated with RYGB 48, suggesting a role for this gene in mediating some of the effects of RYGB. While the effect of RYGB on weight loss has been shown by many studies, the resulting impact of surgery on the lean tissue mass has been inconsistently reported, with some studies showing a decrease, others an increase and yet others unchanged mass compared with sham‐operated controls 47, 49, 50. In contrast, a reduction in adiposity is consistently reported and is sustained over a long period of time, even when the effect of surgery on energy intake has been lost, with intake normalizing or increasing beyond sham‐operated controls 47, 51. The reversal of energy intake in this way may be related to a sustained reduced adipose tissue mass and associated signalling to the hypothalamus, possibly overriding the effect of surgery on the gut and associated hormonal (GLP‐1 and PYY) and bile acid signalling to the central circuits. Indeed, hypothalamic expression of NPY and agouti‐related protein (AgRP) has been shown to increase following long‐term RYGB 50, indicating an orexigenic signalling in the hypothalamus, despite the changes in the gut‐derived anorexigenic signalling, leading to the differential regulation of adipose tissue mass (which decreases) and energy intake (which normalizes). The surgical procedure also causes hypertrophy of the ROUX and common limbs 52, 53, despite reduced appetite 52 and intestinal energy assimilation in humans 54 (Table 1). This indicates a potential link between energy deficit, fat loss, hypothalamic neuropeptide gene expression and intestinal growth. Importantly, the data from RYGB suggest that the energy deficit is signalled via a reduction in white adipose tissue mass, because this tissue shows the most consistent reduction in response to RYGB. Because incoming nutrients are reduced in this situation, the stimulation of the intestinal growth is independent of elevated food consumption, and hence, we can reject model three (Fig. 1) as an explanation for these data. It is important to note however that the nutrient absorptive capacity appears to be retained or even elevated following surgery despite reduced nutrient passage through the gut (because of reduced appetite), as highlighted by the finding that surgically isolated middle part of the python's intestine devoid of contact with luminal nutrients (compared with corresponding intact intestine) shows increased absorptive capacity for L‐leucine and L‐lysine when analysed *in vitro* (Table 1) 13. Moreover, the absorptive capacity of the intestine for glucose uptake can be further enhanced by combining surgery with cold exposure (Table 1) 55.

### Lactation

This energetic challenge provides further insight into the link between adipose tissue‐associated energy loss and intestinal growth. Notably, lactation involves large increases in energy intake and intestinal growth on a background of reduced adipose tissue mass and lowered leptin levels 56, 57. At the hypothalamic level, lactation reduces the expression of POMC and increases the expression of AgRP and NPY in the ARC and increases the expression of NPY in the dorsomedial hypothalamus 58, 59.

The tremendous growth of the intestine during lactation in the rat 60, 61, mouse 62 and other small rodents 56, 63 appears to serve to maintain the absorption efficiency relatively constant in the face of the increase in food intake 57, 64 (Table 1), which may reach four to five times the intake of non‐breeding females 65. Although in general the changes in the alimentary tract seem to serve to sustain assimilation efficiency constant, in some situations, the energy absorption/assimilation efficiency has been reported to change significantly. For example, hamsters show an increased efficiency, while voles show either no change (in 30°C vs. 20°C temperature) or a decrease (upon shaving) (Table 1). In mice, while exposure to cold or shaving does not affect the energy assimilation efficiency of lactating mothers, limiting the access to the cold environment only for lactating mothers appeared to increase this parameter (Table 1). These data highlight the tremendous capacity of the intestine to cope with the elevated intake during lactation and when subjected to additional energetic challenges, which is also reinforced by *in vitro* data showing a greater glucose uptake capacity of intestinal segments in lactating mice when suckling a larger number of pups (Table 1). At the same time that lactating individuals expand their alimentary tract and associated organs like the liver and pancreas to cope with elevated food intake, and their levels of body fat decrease [reviewed in 66]. In some cases, this withdrawal of energy makes a substantial contribution to the total demands of lactation [e.g. in the cotton rat Sigmodon hispidus 67], yet in many small rodents, the contribution is trivially small [calculations in MF1 mice suggest for example that the loss of fat in lactation contributes only about 3% of the total energy demand 66]. It has therefore been suggested that reduced levels of adipose tissue in lactation may serve directly to reduce the production of adipokines, which stimulate the pathways in the hypothalamus that promote food intake 66. This is supported by the fact that provision of exogenous leptin during lactation reduces food intake in rats 68 and Brandt's voles (Lasiopodomys brandtii), and this latter reduction is linked to impacts on the major neuroendocrine levels in the hypothalamus (leptin supplementation reducing NPY and AgRP and stimulating POMC) 69. In addition to stimulating food intake via reduced leptin levels, this reduction of adipose tissue may then also serve to stimulate the prodigious intestinal growth during lactation. Unfortunately, in neither of these leptin repletion studies was the size of the gut measured. The patterns in lactation at room temperature when compared with non‐breeding animals are consistent with all three of the models in Fig. 1.

Modulations of the gastrointestinal tract during lactation in small rodents also vary in relation to the litter size 56, 64, 70, 71, 72. For example, in striped hamsters (*Cricetulus brabensis*), these changes are correlated with changes in food intake, digestive efficiency and gene expression of AgRP in the hypothalamus 56. However, in this situation, the levels of POMC and CART were unchanged despite the large differences in food intake, and trends in both NPY and leptin levels were not significant 56. Given the trends, however, this could be a power issue at the low sample size. In MFI mice, where litters were experimentally manipulated, the small and large intestines were larger in mice raising larger litters, and this was inversely related to the size of the fat stores 71, but in that study, hormone levels and neuropeptide gene expressions were not measured. In voles, litter size is related to food intake, but this is not linked to variations in body fatness nor circulating leptin levels 72. More work is needed to see how litter size effects on food intake in lactation are linked to changes in morphology, circulating hormone levels and gene expression in the hypothalamus.

When lactating mice and other small rodents are exposed to hot conditions (30°C), their food intake declines below that observed at room temperature (21–23°C) 73, 74. Note that we use the term ‘hot’ for 30°C here, despite this being often cited as thermoneutral for mice because this was the term used in the original papers. This is associated with a reduction in the size of the small and large intestine and the caecum 74. Changes in the levels of body fat are less routinely reported in such studies, and the results are inconsistent. In lactating MF1 mice, fat levels are not significantly different between hot conditions and room temperature 75. However, in lactating Brandt's voles 74, body fat contents were increased in the hot conditions. These latter observations are consistent with all three models linking fat content to intestinal growth (Fig. 1).

When lactating mice are additionally exposed to the cold (5°C to 8°C), they further elevate their intake but sustain their absorption efficiency constant 70, 76 (Table 1). This is linked to additional growth in the small intestine above that observed in lactation at room temperature 76. This effect on the tissue is also replicated in other species such as Brandt's voles 77 and in deer mice (Peromyscus maniculatus) 63. As with the response to hot conditions in lactation, the effects on body fat content are however less often measured and less consistent. In lactating mice exposed to the cold, there is a large increase in body fat relative to those housed in warmer conditions 75. This occurs simultaneous to the further expansion of the alimentary tract 75 and hence is inconsistent with models 1 and 2 in Fig. 1, and in this case, the expansion of the tract may be driven directly by the elevated food intake. In contrast, in Brandt's voles and Mongolian Gerbils, there was a further reduction in stored fat that accompanied the expansion of the alimentary tract 77, 78, and in voles, this was linked to lower leptin levels 77. These latter responses are consistent with all three models in Fig. 1, but the responses of mice indicate regulation of intestinal growth at different temperatures in lactation may be independent of the size of the fat stores and directly stimulated by the level of food intake.

### Dietary whey proteins

**Figure 2:**
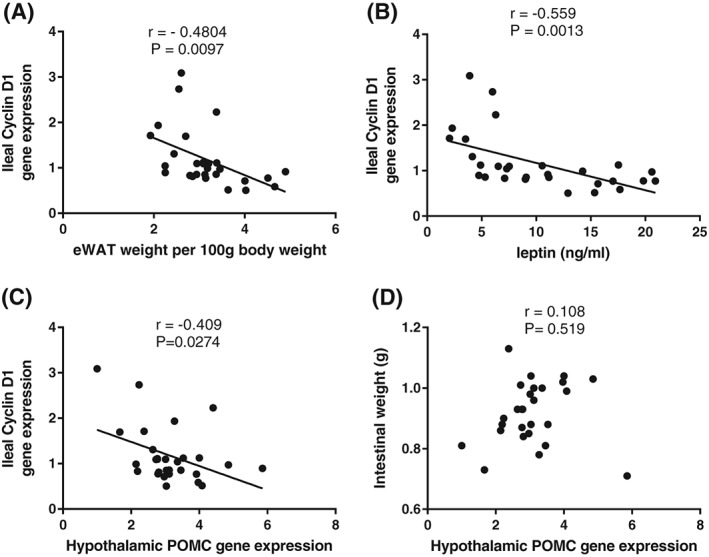
A significant negative correlation exists between the expression of cyclin D1 gene in the ileum and (A) epididymal adipose tissue weight (eWAT), (B) plasma leptin and (C) hypothalamic expression of the pro‐opiomelanocortin (POMC) in mice. This contrast with a lack of a correlation between intestinal weight and hypothalamic expression of POMC (D). Data obtained from a previous study 83, where mice were fed 20% energy whey protein isolate or casein as protein sources along with 35% energy (high) or 7% energy (low) sucrose for 17 week time period (four dietary groups each with *n* = 8).

Milk contains casein and whey proteins; the latter include serum albumin, lactoferrin and alpha‐lactalbumin 79. Because the bovine form of the proteins has been shown to cause an energy deficit in both humans and rodents 80, 81, reflected primarily in reduced white adipose tissue 82, we explored the underlying mechanism and showed that intake of whey protein isolate (WPI) caused a reduction in the ileal expression of fatty acid transport protein 4 (FATP4), glucose transporter 2 (GLUT2) and amino acid transporter (SLC6a19) in mice compared with controls fed casein 83. In addition, the WPI challenge reduced the proportion of the gut microbiota known to be involved in harvesting energy in the gut 83. These changes, coupled with a reduced intestinal fatty acid absorption observed previously in humans, increased fat excretion in mice and increased absorption of specific amino acids in humans (Table 1) 80, 84, suggested a modulated effect of whey proteins on nutrient absorption through the intestine, which possibly accounts for the depleted epididymal white adipose tissue weight and reduced plasma leptin observed in the WPI fed mice 83. Importantly, the mice compensated for this energy deficit by increasing energy intake. This was reflected at a molecular level by changes in the hypothalamic hunger signalling pathways, as the POMC expression was reduced and the NPY expression increased. In the ileum, cyclin D1 gene expression was also increased 83. In the same study, we further showed that the reduction in the adipose tissue mass was the likely cause of the increased cyclin D1 expression in the ileum (rather than energy loss in the intestine) by testing the effects of sucrose content. Notably, reducing the sucrose from 35% to 7% (energy) increased energy expenditure irrespective of the protein source. In the WPI fed mice, this did not further alter the intestinal nutrient transporters or the gut microbiota but further reduced the adipose tissue mass. Concurrently, ileal cyclin D1 gene expression was further increased 83. In fact, as shown in Fig. 2, cyclin D1 gene expression correlated with the change in adipose tissue mass (Fig. 2A), plasma leptin level (Fig. 2B) and hypothalamic POMC expression (Fig. 2C). This correlation was specific to cyclin D1 expression, as POMC expression did not correlate with the intestinal weight (Fig. 2D) nor was there a correlation between other neuropeptides (NPY and ghrelin) and cyclin D1 expression. While these data strengthen the argument that a pathway exists linking the adipose tissue, the hypothalamus and the intestine, which then affects energy assimilation, the lack of a correlative change with the intestinal weight further suggests that other inputs are needed to regulate intestinal growth during adipose tissue‐associated energy loss. In a multiple regression analysis including the gene expression levels of the neuropeptides in the hypothalamus, only leptin receptor expression was related to circulating leptin and the expression levels of intestinal cyclin D1 (*P* < 0.001). This strongly suggests that the impact of adipose tissue withdrawal and reduced leptin levels on intestinal growth acts via the brain (model one: Fig. 1) and not directly (model two: Fig. 1).

### Calorie restriction

Using 0% to 40% CR in 10% increments, it has been shown that there is a correlated decrease in adiposity 85 and plasma leptin 86 in mice. The latter in turn was correlated with graded increase in expression of NPY and AgRP and reduced expression of CART and POMC in the hypothalamus 87. As in the animal models detailed earlier, the graded CR also increased gut size 85. The increased energy assimilation efficiency in this study was modest [approximately 2% 85] and similar to that reported in rats 88, 89. However, this increase was not observed in another study of rats 90, while in a study of non‐human primates under CR, there was a decreased energy assimilation efficiency 91 (Table 1). The variability in nutrient absorption may be related to factors influencing nutrient delivery to the intestine under CR, namely, the type of nutrients provided in the CR diet, extent of energy restriction and its duration. To directly assess the nutrient absorptive capacity of the intestines from CR animals, Ferraris *et al*. 92 and Casirola *et al*. 93 incubated the intestinal regions with define content of radiolabelled nutrients *in vitro* and showed that CR substantially increases the intestinal absorption capacity for D‐glucose, D‐fructose, L‐proline and L‐glutamine (Table 1). Under CR, the intestinal growth occurred when the exogenous nutrient (energy) supply was reduced, suggesting that elevated energy intake is not an essential component of the tissue growth response, consequently allowing us to reject model three in Fig. 1. In this regard, it is interesting that CR increases the number of paneth cells and stem cells in the crypts and their capacity to generate other types of intestinal cells 23. This is possible because of a crucial functional link between the paneth and stem cells as in isolation; neither cell type possessed regenerative capacity 23. The communication pathway involves a differential activation of the mTOR associated complex 1 (mTORC1). Notably, CR reduces mTORC1 activity in the paneth cells, causing the production and release of the paracrine effector, cyclic ADP ribose. This in turn stimulates the adjoining stem cells, increasing the activity of AMPK, leading to protein synthesis 94. Because the crypt cells are in close proximity to the circulating nutrients 15, in particular free fatty acids that are increased in circulation by CR 95, the crypt cells would thus be able to access these energy sources to regenerate, following stimulatory inputs received from the hypothalamus conveying signals related to mobilized fat reserves in the adipose tissue.

## Potential candidates in the adipose–hypothalamic–intestinal signalling pathway

Several pieces of evidence provide clues as to the direction of the pathway and its components. Notably, the adipocyte‐derived hormone leptin regulates hypothalamic neuronal activity linked to energy balance regulation either directly by crossing the blood–brain–barrier 96 or by stimulating the vagal afferent neurons 97. Interestingly, a third potential route of signalling directly from the adipose tissue has been suggested based on viral tract tracing techniques. This work shows that there are sensory neuronal projections from the adipose tissue to hypothalamic nuclei such as the ARC, ventromedial hypothalamus and the dorsomedial hypothalamus 98. The functionality of this pathway was demonstrated by micro‐injection of leptin into the WAT, which increased firing rate of the adipose‐associated sensory neurons 99. One could then extrapolate this functional link to include the intestine based on the discovery that lesions confined to the ARC increased fat absorption 100, while lesions of the ventromedial hypothalamus caused growth of the intestine 101, possibly involving the dorsal motor nucleus of the vagus and associated vagal efferent neurons linked to the control of intestinal functions.

### Adipose‐derived factors

As detailed earlier, leptin is an ideal candidate that could activate the pathway, given that (i) leptin production occurs in proportion to adiposity 86, (ii) it regulates hypothalamic neuropeptide network involved in energy balance regulation [reviewed in 102], (iii) blockade of leptin signalling with *ob* and *db* gene mutations, increases intestinal weight 24, 103 and nutrient absorption 104 and (iv) a correlative link exists between plasma levels of leptin and hypothalamic levels of the leptin receptor and cyclin D1 expression in the intestine (Fig. 2B).

It is also noteworthy that there are also data suggesting an opposite role for leptin in intestinal nutrient absorption. For instance, leptin signalling has been shown to increase GLUT2 and GLUT5 as well as di‐peptide transporter (PepT1) expression in the intestinal cells in the apical side 105, 106; this implies that reduced leptin signalling, as expected with depleting adipose tissues, would reduce nutrient absorption through the intestine, contrary to the aforementioned data presented. The discrepancy could be related to the fact that leptin is produced by adipocytes and gastric cells 107, with intestinal cells experiencing the effect of leptin from the apical side (from leptin in the lumen) as well as from the basolateral side (possibly from leptin signalling via the hypothalamic route). To assess the direct effects of leptin on intestinal cellular activity, Tavernier *et al*. generated mice with targeted deletion of the leptin receptor in the intestinal epithelial cells 108. In these mutant mice, there were no significant changes in energy intake, body weight and the intestinal expression of GLUT2 compared with wild type controls fed low fat diet. In the intestine, the gene mutation increased the villi height, albeit the intestinal length was unaffected. The activity of the PepT1 transporter was reduced 108. These data suggest that luminal and systemic leptins (possibly via a central route) have diverse effects on the intestinal mechanisms linked to energy absorption, where their coordinate effects may ultimately determine the cellular changes in the intestine with regard to leptin action. A third possibility is that changes in energy content in the adipose tissue are conveyed directly to the intestine by factors other than leptin (model two in Fig. 1). Indeed, there is evidence suggesting this, because mice with defective leptin signalling (*db/db* mutation) show increased cell proliferation in the intestine as well as increased intestinal weight, which was rescued by CR, a regime that is known to significantly reduce body weight in the mutant mice 24. Given that CR in wild type mice increased the size of the alimentary tract 85, whereas CR in obese *db/db* mice reduced the intestinal weight 24, indicate a complex regulation of intestinal growth, involving multiple extrinsic (e.g. nutrients) and intrinsic factors (e.g. leptin). This is further supported by the finding that mice devoid of exogenous nutrient supply (i.e. starvation) and injected with leptin do not alter intestinal weight 109.

### Hypothalamic neuropeptides

Of these, gene knockout studies show that NPY associated Y2 and Y4 receptors increase fat absorption through the intestine 110. Like NPY, hypothalamic ghrelin also has orexigenic effects as demonstrated by the finding that central ghrelin action reduced intestinal cellular apoptosis and increased villi height 111. These data together with the correlational changes shown here between ileal cyclin D1 gene expression and hypothalamic POMC expression (Fig. 2C) 83 suggest a potential role for leptin sensitive hypothalamic POMC, ghrelin and NPY neuropeptides in conveying the signals related to the loss of adipose mass to the intestine, leading to increased intestinal cell proliferation (possibly involving POMC) and reduced cell apoptosis (involving ghrelin) alongside increased fat absorption (involving NPY).

### Intestinal signalling molecules

A key function of the intestine is to absorb nutrients. This is determined by genes encoding nutrient transporters and by tissue growth (cell proliferation and apoptosis). Several pieces of evidence suggest that these two parameters can be regulated independently. The data from our work on whey proteins in the diet showed that dietary WPI reduced the expression of nutrient transporters (GLUT2, FATP4 and SLC6a19) but at the same time increased intestine weight 83. Similarly, the surgically isolated central part of the python intestine devoid of contact with luminal nutrients showed increased nutrient absorptive capacity but unchanged tissue mass 13. This dual mode of regulation (of intestinal nutrient transporters vs. tissue growth) is potentially advantageous in the event the intestinal nutrient transporter system is down‐regulated, as with WPI feeding, because then the resultant depletion of the adipose tissue can signal to the intestine to promote tissue growth to assimilate more nutrients via passive uptake. Key intestinal signalling molecules involved in this process include ornithine decarboxylase 112, mTOR 113 and cyclin D1, acting alongside nutrient‐derived energy to fuel cell division and tissue growth. The ability of the intestinal cells to utilize luminal and circulating nutrients for tissue growth is also another advantage because it would aid the improvement in circulating lipid and glucose levels as seen in obese and diabetic patients subjected to the aforementioned energetic challenges 114, 115, 116, 117, 118, 119.

## Conclusion

Living organisms are rarely in energy homeostasis because they face periods of energy deficit (from food shortage or from increased demands) and periods of excess energy availability, when food may be consumed in excess of immediate demands to build‐up fat reserves. Investing energy to promote the growth of the intestine may allow animals to maximize their energy absorption, allowing them to meet elevated demands or bring themselves back into energy balance. This energy investment in the intestine is fuelled by external (food consumed) and internal nutrient sources (circulating nutrients), which promote epithelial and crypt associated cell division. We emphasize here that depletion of white adipose tissue may result in production of signals (probably but not exclusively including leptin), which signal to the hypothalamus to elevated food intake and stimulate intestinal growth. The available data suggest that white adipose tissue depletion is more likely to act via the brain than directly on the intestine.

## Conflict of interest statement

No conflict of interest was declared.
